# Induction of Lipocalin2 in a Rat Model of Lung Irradiation

**DOI:** 10.3390/ijms17050637

**Published:** 2016-04-28

**Authors:** Sadaf Sultan, Shakil Ahmad, Margret Rave-Fränk, Ihtzaz Ahmed Malik, Clemens F. Hess, Hans Christiansen, Silke Cameron

**Affiliations:** 1 Clinic for Gastroenterology and Endocrinology, University Medical Center Göttingen, Robert-Koch-Str. 40, D-37075 Göttingen, Germany; drsadafsultan@gmail.com (S.S.); shakil.ahmad@med.uni-goettingen.de (S.A.); i.malik@med.uni-goettingen.de (I.A.M.); 2 Clinic for Radiotherapy and Radiation Oncology, University Medical Center Göttingen, Robert-Koch-Str. 40, D-37075 Göttingen, Germany; mfraenk@med.uni-goettingen.de (M.R.-F.); cfhess@med.uni-goettingen.de (C.F.H.); Christiansen.Hans@mh-hannover.de (H.C.)

**Keywords:** lung irradiation, lipocalin2, upper and lower part of the liver

## Abstract

Previously, we showed that lipocalin2 (LCN2) serum levels increased after liver irradiation and during acute-phase conditions. Here, we evaluate LCN2 expression and serum levels after single-dose lung irradiation with 25 Gy, percutaneously administered to the lung of randomly-paired male Wistar rats. Due to the concave anatomy of the lung recesses, the irradiation field included the upper part of the liver. No rat died due to irradiation. In control tissue, lung immunohistochemistry showed a high constitutive expression of LCN2+ granulocytes. LCN2 mRNA levels in lung tissue increased up to 24 h (9 ± 2.3-fold) after irradiation. However, serum LCN2 levels remained undetectable after lung irradiation. LCN2 expression in the upper part of the liver increased up to 4.2-fold after lung irradiation, but the lower liver showed an early decrease. Acute-phase cytokines (IL-1β and TNF-α) showed a significant increase on transcript level in both lung and upper liver, whilst the lower liver did not show any considerable increase. In conclusion, constitutive expression of LCN2 in local immune cells demonstrates its local role during stress conditions in the lung. The absence of LCN2 in the serum strengthens our previous findings that the liver is the key player in secreting LCN2 during stress conditions with liver involvement.

## 1. Introduction

Lipocalin2 (LCN2) is a small protein of 25 kDa, which was found to be associated with human neutrophil gelatinase (syn. neutrophil gelatinase associated lipocalin, NGAL) [1]. LCN2 binds to bacterial siderophores and protects the iron of the body during bacterial infection, an important response within the innate immune system [2]. Previously, we described LCN2 as a major positive acute phase protein in rats [3].

LCN2 expression has been demonstrated in neutrophils, the bone marrow and in tissues that are prone to the attack of microorganisms, *i.e.*, lung, trachea, stomach, colon and salivary gland, denoting its role in inflammatory conditions [4]. Accordingly, several other studies demonstrated an upregulation of LCN2 expression in epithelial cells of human and murine tissues during different inflammatory conditions, such appendicitis, inflammatory bowel disease and diverticulitis [5,6]. Furthermore, LCN2 elevation has been shown in systemic inflammatory states, such as sepsis or endotoxin shock [7]. Overexpression of LCN2 has also been described in several types of human cancer cells, such as colorectal, bone marrow and pancreatic cancer [6,8,9]. In this context, it has also been demonstrated that LCN2 promotes invasion, migration and pulmonary metastasis [10].

The lung is a vital organ and a major route through which a number of pathogens can enter the body and cause inflammation [11]. Lung cancers are increasingly treated by radiotherapy, especially with the advent of respiratory guided radiotherapy and the improvement of stereotactic techniques. It may be combined with chemotherapy in patients who are not eligible for surgery [12].

We have previously shown an increased LCN2 expression both at the transcriptional and translational level in irradiated rat liver, where LCN2 serum levels showed a strong increase after liver irradiation [13]. As non-operable lung cancers are increasingly treated with radiation, we decided to irradiate the lung, in order to determine the role of LCN2. Gene expression of LCN2 was studied in normal, irradiated lung tissue and serum. Due to the concave anatomy of the lung, the upper part of the liver was within the radiation field during lung irradiation. To further compare LCN2 gene expression in both liver parts, the liver tissue was separated into the upper part (within the radiation field) and the lower part (outside of the radiation field). Besides LCN2, the expression of acute phase cytokines was evaluated in the lung, the upper and lower part of the liver.

## 2. Results

### 2.1. Serum Lipocalin2 (LCN2) Concentration after Lung Irradiation

Serum LCN2 was not detected after lung irradiation, neither by ELISA, nor by Western blot. Liver homogenate was used as a positive control for both, ELISA and Western blot analysis.

### 2.2. LCN2 Transcript Expression in Lung Tissue

**Figure 1:**
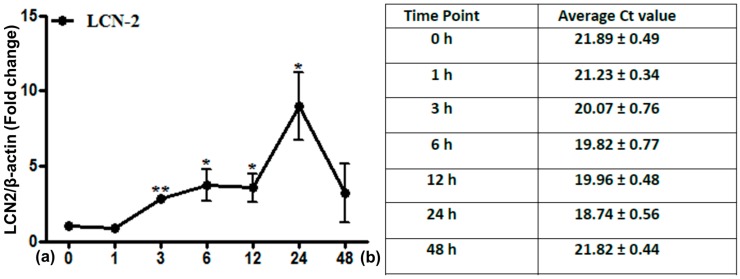
Fold change of mRNA expression of Lipocalin2 (LCN2) in irradiated lung tissue at time points from 1 to 48 h. Data are given relative to normal sham irradiated control rats (**a**). The results were normalized to the housekeeping gene β-actin, experimental errors are shown as ±SEM (* *p* ≤ 0.05, ** *p* ≤ 0.005, *n* = 3). Ct values are depicted in (**b**).

Analysis of LCN2 mRNA expression from irradiated lung tissue showed a relatively high constitutive expression (Figure 1). LCN2 levels increased further after irradiation, reaching significance at 3 h and a maximum at 24 h with a nine-fold increase (±2.25), followed by a decrease thereafter.

### 2.3. LCN2 Protein Expression in Lung Tissue

**Figure 2:**
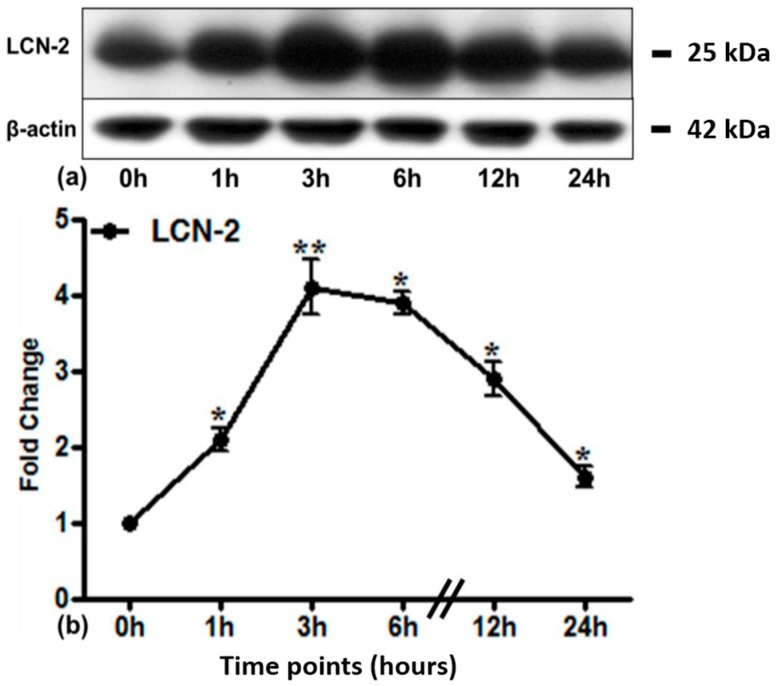
Western blot analysis of LCN2 (25 kDa) protein in the lung of control and irradiated animals at different time points. β-actin (42 kDa) was used as the loading control (**a**). Densitometric analysis of Western blots was performed to show the changes in protein expression of LCN2 (**b**). Results are shown as fold change ± SEM (* *p* ≤ 0.05, ** *p* ≤ 0.005, *n* = 3).

Accordingly, there was a strong constitutive expression of LCN2 protein in lung tissue (Figure 2a). Irradiation of the lung showed a further and significant increase of LCN2 protein expression after 1 h, reaching a maximum at 3 h (4.2 ± 1.10-fold, densitometric analysis; Figure 2b), whilst maintaining significance up to 24 h.

### 2.4. Histochemistry and LCN2 Immunofluorescence Staining in Sham and Irradiated Lung Tissue

**Figure 3:**
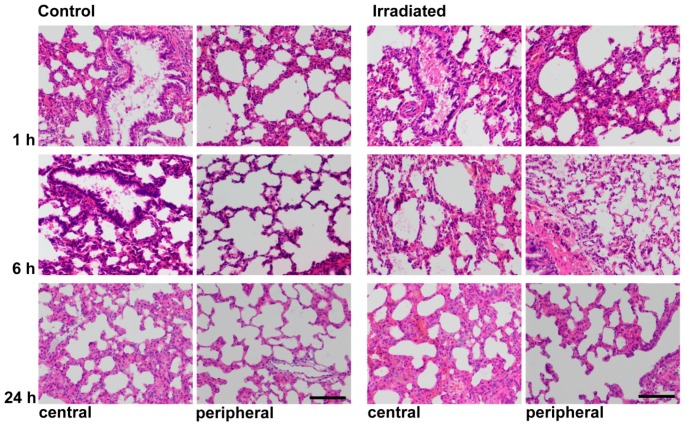
Hematoxylin-eosin staining of central and peripheral areas of the lung at 1, 6 and 24 h of sham irradiated rats (control) and after single-dose irradiation with 25 Gy (original magnification 200×, scale bar = 100 μm).

Histochemical Hematoxylin-eosin (HE) staining of central and peripheral areas of the lung showed blood congestion in central parts of the lung and consecutive thickening of the alveolar walls at 1 h after irradiation. At 6 h, the alveolar lining was thinner with almost complete reconstitution at 24 h (Figure 3).

**Figure 4:**
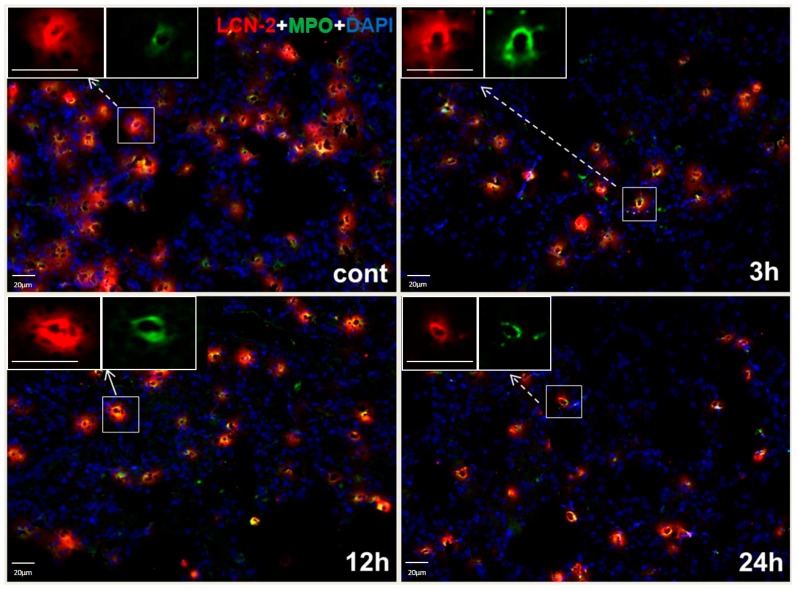
Double immunofluorescence staining of LCN2 and myeloperoxidase (MPO)-positive cells in representative sections of lung tissue for control rats (cont) and 3, 12 and 24 h after irradiation. Cryosections were stained with anti-LCN2 (red) and anti-MPO (green), followed by fluorescence immunodetection. Counterstaining of the nuclei was done with DAPI (blue) (original magnification 200×, scale bar = 20 μm).

Double-immunofluorescence staining of the lung showed LCN2 protein in myeloperoxidase-positive (MPO+) granulocytes (Figure 4). At 3, 12 and 24 h after irradiation, immunofluorescence staining showed no increase of LCN2-positive granulocytes.

### 2.5. Gene Expression of Different Acute Phase Cytokines in Sham and Irradiated Lung Tissue

**Figure 5:**
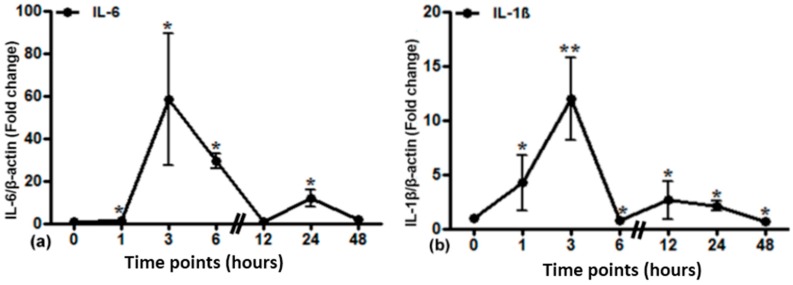

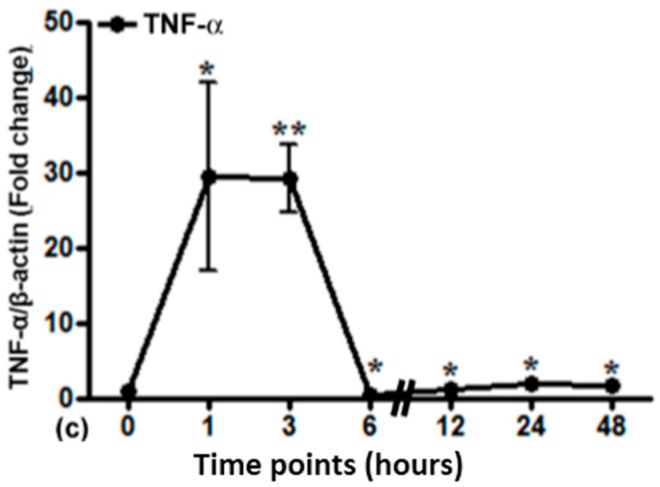
Relative mRNA expression of acute phase cytokines (IL-6 (**a**), IL-1β (**b**) and TNF-α (**c**)) in irradiated lung tissue. The results were normalized to β-actin as the housekeeping gene. Experimental errors are depicted as ±SEM (* *p* ≤ 0.05, ** *p* ≤ 0.005, *n* = 3).

Local expression of different cytokines after lung irradiation was determined by RT-PCR analysis (Figure 5). IL-6 mRNA expression was significantly elevated up to 60-fold at 3 h. IL-1β gene expression was significantly elevated at 1 h and reached its maximum at 3 h (12-fold). TNF-α gene expression reached a maximum of up to 30-fold at 1 h with a plateau until 3 h and a consecutive decrease.

### 2.6. LCN2 Transcript Expression in the Upper and Lower Part of the Liver

**Figure 6:**
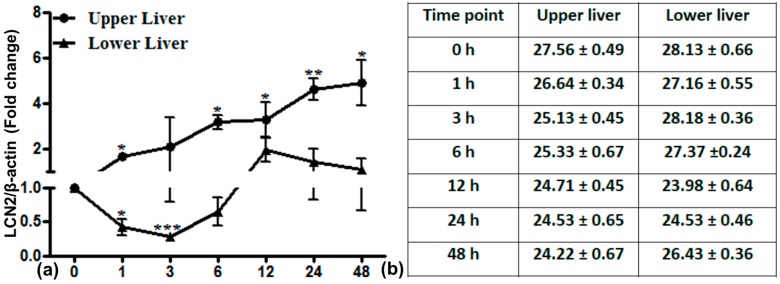
Relative mRNA expression of LCN2 in the upper and lower part of the liver after lung irradiation. Data are given relative to normal sham irradiated control rats (**a**). The results were normalized to β-actin as the housekeeping gene. Experimental errors are depicted as ±SEM (* *p* ≤ 0.05, ** *p* ≤ 0.005, *** *p* ≤ 0.0005, *n* = 3). Ct values are shown in (**b**).

LCN2 expression was significantly higher in the directly irradiated upper part than in the lower part of the liver (Figure 6). In the upper part, LCN2 expression started to increase directly after irradiation (1 h) and reached a maximum of up to five-fold (±1.01) within 48 h. The lower part of the liver showed a decrease of LCN2 expression from 1 to 6 h, which was significant at 1 and 3 h with recovery later on.

### 2.7. LCN2 Protein Expression in the Upper and Lower Part of the Liver

**Figure 7:**
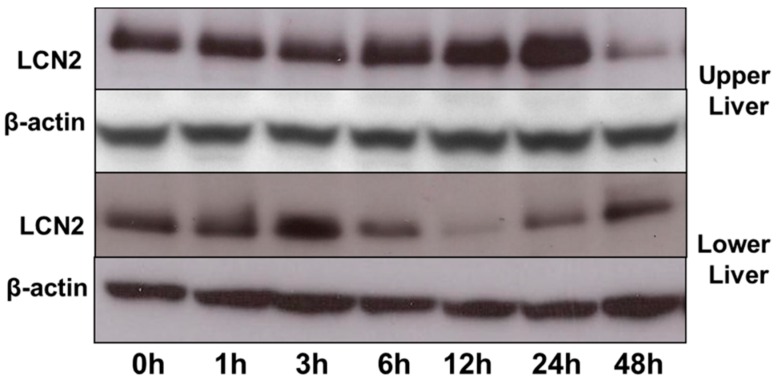
Western blot analysis of LCN2 (25 kDa) protein in the upper and lower part of the liver. β-actin (42 kDa) was used as the internal loading control.

LCN2 protein expression of the upper and lower part of the liver further confirmed our results of LCN2 mRNA expression (Figure 7). An increase in LCN2 protein expression until 24 h was seen in the upper part of the liver after lung irradiation. The LCN2 protein expression from the lower part of the liver showed a decrease, especially from 6 to 24 h (a short delay when compared to mRNA expression).

### 2.8. LCN2 Immunostaining of Liver Tissues after Lung Irradiation

**Figure 8:**
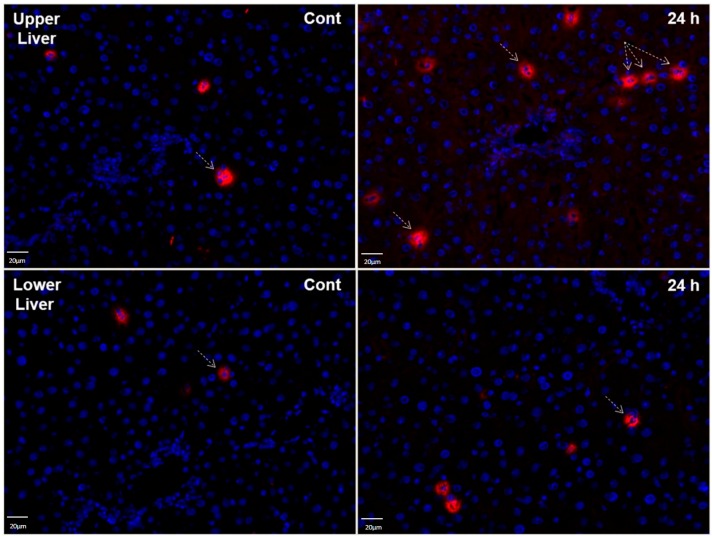
Immunofluorescence staining of LCN2 in tissue sections of upper and lower parts of the liver from sham irradiated control rats (Cont) and 24 h after irradiation. Sections were stained with anti-LCN2 (red, exemplary LCN2 positive cells are pointed out with arrows) followed by a fluorescence immunodetection. Counterstaining of the nuclei was done with DAPI (blue) (original magnification 200×).

Immunofluorescence staining of the upper part of the liver showed a slight increase of LCN2-positive immune cells within 24 h after irradiation as compared to sham-irradiated controls. Changes in LCN2-positive cells in the lower liver were negligible (Figure 8).

### 2.9. Gene Expression of Different Cytokines in the Upper and Lower Part of the Liver after Lung Irradiation

**Figure 9:**
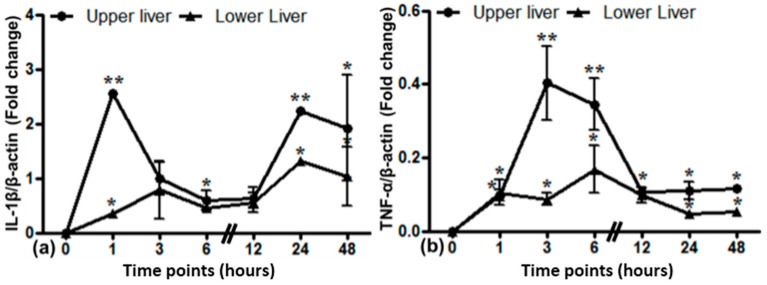
Relative mRNA expression of acute phase cytokines IL-1β (**a**) and TNF-α (**b**) in the upper and lower liver from the irradiated lung experiment. The results were normalized to β-actin as the housekeeping gene. Experimental errors are depicted as ±SEM (* *p* ≤ 0.05, ** *p* ≤ 0.005, *n* = 3).

Local gene expression of different cytokines in the upper and lower part of the liver was evaluated by PCR analysis (Figure 9). IL-1β expression was increased in the upper part of the liver within 1 h after lung irradiation (up to 2.6-fold) followed by a sudden decrease. In the lower liver, there was no such peak in IL-1β expression when compared to the upper part of the liver. TNF-α expression showed an early, but small increase in the upper liver with a maximum at 3 h (0.4-fold). In the lower liver, no major differences were seen.

## 3. Discussion

LCN2 is expressed at a low steady-state level unless cellular stress occurs [3]. The lung airways are a unique environment, where an adequate response of the immune system is required. The upper airways have to bear a permanent exposure to environmental effects and commensal organisms [11]. In the lung, we showed a strong constitutive LCN2 gene expression by RT-PCR, Western blot and immunostaining, which was further upregulated by irradiation. It has been suggested that LCN2 upregulation during stress conditions has anti-inflammatory functions, thus preventing ongoing tissue damage [14].

In order to prevent airway occlusion and destruction, *i.e.*, during infections, the immune response in the lung is induced rapidly , but excessive inflammation has to be prevented, as it would lead to airway destruction and airway occlusion with compromised gaseous exchange [15]. An important role of LCN2 in inflammation has been shown in adipocytes and macrophages [16], as well as granulocytes [17]. Furthermore, Warszawska *et al.* showed that LCN2 attenuated the early inflammatory response in a STAT-3-dependent manner by inducing IL-10 formation [18].

Our current results underline the local role of LCN2 expression in view of normal external stimuli and during stress response induced by irradiation. After lung irradiation, LCN2 gene expression increased early and significantly. LCN2 positivity co-localized with MPO+ neutrophil granulocytes, which supports its role during oxidative stress conditions [19]. The increase of LCN2 gene expression within lung tissue after irradiation, however, was not mirrored by an increased number of granulocytes. The early increase of LCN2 expression could, on the one hand, be explained by granulocyte activation. On the other hand, it has been suggested that epithelial cells are a source of LCN2 under stress conditions [5,6,13]. Instead of being secreted into the blood stream, LCN2 would be secreted into the alveoli. In this setting, irradiation leads to damage of granulocytes and epithelial cells (reflected by thinner alveolar walls at 6 h after irradiation) and, as a consequence, local release of LCN2. This would further explain the failure to detect LCN2 in the serum, since immunohistochemistry would not capture released LCN2 as it is a secretory protein. It also explains that LCN2 protein is elevated early (after 1 h) in whole tissue homogenate with elevation up to 24 h.

We showed earlier that LCN2 expression might not only be triggered by radiation itself, but also by radiation-induced cytokine activation [13]. For lung irradiation, we could now confirm these data and show that transcript expression of pro-inflammatory cytokines precedes LCN2 upregulation.

For systemic LCN2 concentrations, the liver seems to play a major role. As we showed earlier, whole liver irradiation led to a significant release of LCN2 into the serum [13]. Within the liver, hepatocytes are the key source of LCN2 [13,20]. Even though hepatocytes, Kupffer cells and myofibroblasts produce LCN2 [13], it has been shown, using conditional LCN2 knockout mice, that if LCN2 is knocked out in hepatocytes, no serum levels of LCN2 were found [21]. This further confirms our findings after lung irradiation, in which we show a lack of serum LCN2 release. Interestingly, irradiation of the small upper part of liver, which was within the irradiation field in the current study, was not enough to produce detectable amounts of LCN2 in the serum. Even for the liver, a volume effect might be important for the release and detection of LCN2 serum levels. Such a volume effect has been observed for the development of clinical radiation-induced liver diseases (RILD) [22]. Radiation-induced lung injury (RILI) with radiation pneumonitis and pulmonary fibrosis is a later event and probably needs multiple radiation events [23].

In our irradiation model of 25 Gy, lung anatomy remained intact within the examined time-frame, with slight swelling of the alveolar walls. We could show that expression of LCN2 only seems to be sufficient to maintain a local response and does not lead to systemic LCN2 detection. It might be possible that local LCN2 upregulation triggers anti-inflammatory pathways maintaining tissue homeostasis and prevent tissue damage, as suggested by Zhang *et al.* [16]. The immunosuppressive role of LCN2 [24] has also been suggested to facilitate tumor growth [25]. As LCN2 expression has been reported by several types of cancer [6,8,9] and is induced in hypoxic states, it is reasonable to argue that iron sequestration by LCN2 might promote cell survival and tumorigenesis [25].

Future studies, for instance with a conditional LCN2 knockout mouse model, would help to understand the role of LCN2 during and after irradiation damage. Consequently, further studies in tumor models would be needed.

## 4. Material and Methods

### 4.1. Animals

Adult male Wistar rats with a body weight of 180–200 g were obtained from Harlan-Winkelmann (Borchen, Germany). They were kept in our animal facility at a 24-h day and night cycle with an ambient room temperature of 20 °C–25 °C and free access to water and standard rat food. Each rat consumed about 12–15 g of food and 12–25 mL of water and gained 30–40 g weight/week. All animal experiments were performed in accordance to the institutional policies and the recommendations for care and use of laboratory animals. The treatment of rats was approved by the local ethics committee of the University Medicine of Goettingen and the public authority on animal welfare. Each time point was studied in triplicate with two sham irradiated animals as controls for each experimental group.

Experiments were part of the Ph.D. thesis of the first author (Sadaf Sultan) and carried out as described in [26], which we briefly summarize here.

### 4.2. Irradiation of Rat Lung

**Figure 10:**
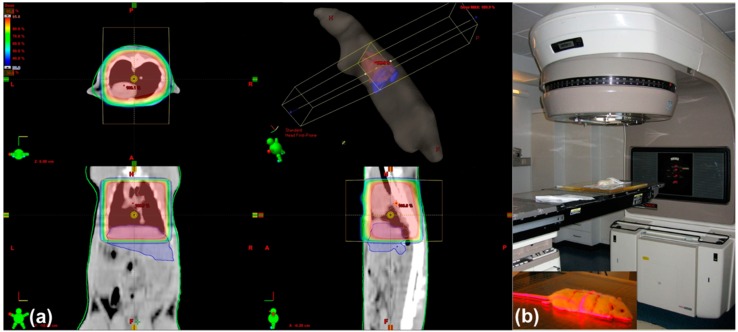
Planning CT-scan and dose distribution (**a**). The area for lung irradiation was marked on the fur of the rats. This area was then irradiated in a two-field technique (**b**).

According to our previously-published model of single-dose irradiation [27], we here describe a lung irradiation model, where a single dose of 25 Gy was delivered to the lung using an antero-posterior/postero-anterior (AP/PA) radiation technique [26]. Exactly as in the previous protocol [26,27], dose distribution was calculated following a planning CT scan (Somatom Balance, Siemens, Erlangen, Germany). For this purpose, each rat was anesthetized intraperitoneally with 90 mg/kg ketamine (Intervet, Unterschleißheim, Germany) and 7.5 mg/kg xylazine 2% (Serumwerk Bernburg AG, Bernburg/Saale, Germany). The lung margins were drawn on the fur of the animals. An example for the dose distribution of lung x-radiation is given in Figure 10a. Irradiation of the targeted organ was then done at a dose rate of 2.4 Gy/min with 6 MV photons using a Varian Clinac 600 C accelerator (Varian, Palo Alto, CA, USA) (Figure 10b), resulting in a 25-Gy single dose. Because of the convex anatomy of the lung recesses, the upper portion of the liver was situated in the radiation field, whilst the lower portion of the liver remained outside (Figure 10). Sham irradiated rats served as controls.

Treated animals and sham irradiated controls were sacrificed humanely at 1, 3, 6, 12, 24 and 48 h after irradiation. The lung, the upper (irradiated) and lower (out-of-field) portion of the liver were removed carefully, washed in 0.9% NaCl and stored at −80 °C for later RNA and protein analysis or fixed in formalin for preparation of paraffin blocks. Blood was collected in special serum tubes (Sarstedt Monovette, Nuembrecht, Germany).

### 4.3. Measurement of Serum LCN2 Levels

LCN2 serum concentrations were evaluated by an ELISA kit with 0.5 pg/mL as the detection limit (Bioporto Diagnostics, Kit 046, Gentofte; Denmark).

For further confirmation, immunoblotting was done to detect LCN2 protein in the serum, but no LCN2 was detected after lung irradiation.

### 4.4. RNA Isolation and RT-PCR

Small pieces from lung and both parts of liver tissues were homogenized in TRIzol (Invitrogen, California, Carlsbad, CA, USA), and total RNA was extracted using ultracentrifugation, as described previously [28]. RNA content was measured by a spectrophotometer at 260/280 nm. In the next step, cDNA was synthesized by reverse transcription of isolated RNA utilizing the commercial superscript kit (Invitrogen, Groningen, The Netherlands), according to the manufacturer’s instructions. The targeted genes were amplified in the Step one Plus real-time PCR cycler (Applied Biosystems, Darmstadt, Germany) at 95–60 °C for 40 thermal cycles. SYBR Green UDG reaction master mix (Invitrogen Groningen, The Netherlands) served for relative quantification of cDNA. The primers used to quantify the expression of targeted genes (Invitrogen, Groningen, The Netherlands) are given in Table 1. β-actin was chosen as internal control to normalize gene expression.

### 4.5. Protein Extraction and Immunoblotting

Protein isolation from lung and liver tissues of all experimental rats was done according to our published protocols [3,26,29]. Isolated proteins were quantified by the Coomassie Assay (Pierce, Germany). For immunoblotting, reducing sodium dodecyl sulfate (SDS) polyacrylamide gel electrophoresis (PAGE) was performed as detailed in [30], using β-actin as the internal loading control. After protein transfer onto Hybond ECL nitrocellulose hybridization membranes according to [7,29], the expression of targeted proteins was detected by the ECL Western blotting kit of GE Healthcare (Munich, Germany). Mouse monoclonal anti-LCN2 and anti-β-actin primary antibody dilutions were 1:300 and 1:5000, respectively. Anti-mouse immunoglobulins (1:2000) served as secondary antibodies. The immunoblots were analyzed densitometrically by ImageJ software (NIH, available at: https://imagej.nih.gov/ij/).

### 4.6. Histology and Immunohistochemistry

Formalin-fixed and paraffin-embedded tissue sections of 2 µm were stained with hematoxylin and eosin (H & E) after deparaffinization. For immunohistochemistry, cryostat sections (4 µm) of frozen tissues, lung, upper and lower liver, were cut, briefly dried in air and immediately fixed in ice-cold acetone (−20 °C). After fixation, we incubated the issue sections for 1 h in fetal calf serum (FCS) enclosed in a humidified chamber to inhibit nonspecific binding of antibody during the staining procedure [26,27]. After blocking with FCS, tissue sections were washed in phosphate-buffered saline (PBS) three times for 10 min each. Thereafter, sections were incubated with anti-LCN2 antibody (mouse monoclonal, Novus biological, NBPI-05182) or anti-rabbit myeloperoxidase (MPO) (polyclonal, Dako, A0398) overnight at +4 °C at a concentration of 1:100 and 1:50, respectively. The MPO antigen has been isolated from granulocytes. The antibody thus reacts with neutrophil granulocytes. After overnight incubation, the slides were again washed three times in PBS, followed by incubation at room temperature for 1 h in darkness with the fluorescence-labelled secondary antibody: Alexa Fluor 555 goat anti-mouse or Alexa Fluor 488 goat anti-rabbit (Invitrogen), respectively. Counterstaining of the nuclei was done with diamidino-2-phenylindole (DAPI) (4 µL in 100 mL of PBS). The slides were mounted with Fluoromount-G (0100-01, Southern Biotech, Birmingham, AL, USA).

### 4.7. Statistical Analysis

Statistical analysis of the obtained experimental data was performed by GraphPad Prism 5 and Microsoft Excel 2007 software. PCR-values, *i.e.*, cycle thresholds (*C*_t_) of irradiated rats, were normalized to control values. The change in gene expression was calculated relative to the expression of β-actin. Errors are shown as the standard error of the mean (SEM). Statistical differences were evaluated by Student’s *t*-test. *p* ≤ 0.05 was considered as statistically significant.

## 5. Conclusions

We propose that LCN2 has a local protective role in normal as well as in radiation-induced stress conditions. Elevated LCN2 expression after lung irradiation denotes its role in oxidative stress and inflammation. Tight control of LCN2 expression might be important to prevent the development of epithelial hyperproliferation in inflammatory conditions and lung adenocarcinoma [31]. The absence of LCN2 in the serum after lung irradiation strengthens our previous findings that the liver might be the key player in secreting LCN2 during stress conditions with liver involvement. Further studies are necessary to explore acute phase conditions in the absence of LCN2.

## Figures and Tables

**Table 1:** Gene-specific synthesized forward and reverse primer sequences used for RT-PCR analysis.

Gene (Rat)	Forward 5′–3′	Reverse 5’–3’
LCN2	GGAATATTCACAGCTACCCTC	TTGTTATCCTTGAGGCCCAG
β-actin	TGTCACCAACTGGGACGATA	AACACAGCCTGGATGGCTAC
IL-6	GTCAACTCCATCTGCCCTTCAG	GGCAGTGGCTGTCAACAACAT
IL-1β	TACCTATGTCTGGCCCGTGGAG	ATCATCCCACGAGTCACACAGG
TNF-α	ACAAGGCTGCCCCGACTAT	CTCCTGGTATGAAGTGGCAAATC

## References

[1] Kjeldsen L., Johnsen A.H., Sengelov H., Borregaard N. (1993). Isolation and primary structure of NGAL, a novel protein associated with human neutrophil gelatinase. J. Biol. Chem..

[2] Yang J., Goetz D., Li J.Y., Wang W., Mori K., Setlik D., Du T., Erdjument-Bromage H., Tempst P., Strong R. (2002). An iron delivery pathway mediated by a lipocalin. Mol. Cell.

[3] Sultan S., Pascucci M., Ahmad S., Malik I.A., Bianchi A., Ramadori P., Ahmad G., Ramadori G. (2012). LIPOCALIN-2 is a major acute-phase protein in a rat and mouse model of sterile abscess. Shock.

[4] Cowland J.B., Borregaard N. (1997). Molecular characterization and pattern of tissue expression of the gene for neutrophil gelatinase-associated lipocalin from humans. Genomics.

[5] Alpizar-Alpizar W., Laerum O.D., Illemann M., Ramirez J.A., Arias A., Malespin-Bendana W., Ramírez V., Lund L.R., Borregaard N., Nielsen B.S. (2009). Neutrophil gelatinase-associated lipocalin (NGAL/LCN2) is upregulated in gastric mucosa infected with *Helicobacter pylori*. Virchows Arch..

[6] Nielsen B.S., Borregaard N., Bundgaard J.R., Timshel S., Sehested M., Kjeldsen L. (1996). Induction of NGAL synthesis in epithelial cells of human colorectal neoplasia and inflammatory bowel diseases. Gut.

[7] Sunil V.R., Patel K.J., Nilsen-Hamilton M., Heck D.E., Laskin J.D., Laskin D.L. (2007). Acute endotoxemia is associated with upregulation of lipocalin 24p3/LCN2 in lung and liver. Exp. Mol. Pathol..

[8] Bartsch S., Tschesche H. (1995). Cloning and expression of human neutrophil lipocalin cDNA derived from bone marrow and ovarian cancer cells. FEBS Lett..

[9] Furutani M., Arii S., Mizumoto M., Kato M., Imamura M. (1998). Identification of a neutrophil gelatinase-associated lipocalin mRNA in human pancreatic cancers using a modified signal sequence trap method. Cancer Lett..

[10] Shi H., Gu Y., Yang J., Xu L., Mi W., Yu W. (2008). Lipocalin2 promotes lung metastasis of murine breast cancer cells. J. Exp. Clin. Cancer Res..

[11] Travis S.M., Conway B.A., Zabner J., Smith J.J., Anderson N.N., Singh P.K., Greenberg E.P., Welsh M.J. (1999). Activity of abundant antimicrobials of the human airway. Am. J. Respir. Cell Mol. Biol..

[12] Wagner H. (1998). Radiation therapy in the management of limited small cell lung cancer: When, where, and how much?. Chest.

[13] Sultan S., Cameron S., Ahmad S., Malik I.A., Schultze F.C., Hielscher R., Rave-Fränk M., Hess C.F., Ramadori G., Christiansen H. (2013). Serum Lipocalin2 is a potential biomarker of liver irradiation damage. Liver Int..

[14] Flower D.R. (1996). The lipocalin protein family: Structure and function. Biochem. J..

[15] Reynolds H.Y. (1987). Lung inflammation: Normal host defense or a complication of some diseases?. Annu. Rev. Med..

[16] Zhang J., Wu Y., Zhang Y., Leroith D., Bernlohr D.A., Chen X. (2008). The role of lipocalin2 in the regulation of inflammation in adipocytes and macrophages. Mol. Endocrinol..

[17] Schroll A., Eller K., Feistritzer C., Nairz M., Sonnweber T., Moser P.A., Rosenkranz A.R., Theurl I., Weiss G. (2012). Lipocalin2 ameliorates granulocyte functionality. Eur. J. Immunol..

[18] Warszawska J.M., Gawish R., Sharif O., Sigel S., Doninger B., Lakovits K., Mesteri I., Nairz M., Boon L., Spiel A. (2013). Lipocalin2 deactivates macrophages and worsens pneumococcal pneumonia outcomes. J. Clin. Investig..

[19] Nakazato T., Sagawa M., Yamato K., Xian M., Yamamoto T., Suematsu M., Ikeda Y., Kizaki M. (2007). Myeloperoxidase is a key regulator of oxidative stress mediated apoptosis in myeloid leukemic cells. Clin. Cancer Res..

[20] Ahmad S., Sultan S., Naz N., Ahmad G., Alwahsh S.M., Cameron S., Moriconi F., Ramadori G., Malik I.A. (2014). Regulation of iron uptake in primary culture rat hepatocytes: The role of acute phase cytokines. Shock.

[21] Xu M.J., Feng D., Wu H., Wang H., Chan Y., Kolls J., Borregaard N., Porse B., Berger T., Mak T.W. (2015). Liver is the major source of elevated serum lipocalin-2 levels after bacterial infection or partial hepatectomy: A critical role for IL-6/STAT3. Hepatology.

[22] Dawson L.A., Ten Haken R.K., Lawrence T.S. (2001). Partial irradiation of the liver. Semin. Radiat. Oncol..

[23] Huang K., Palma D.A., IASLC Advanced Radiation Technology Committee (2015). Follow-up of patients after stereotactic radiation for lung cancer: A primer for the nonradiation oncologist. J. Thorac. Oncol..

[24] La Manna G., Ghinatti G., Tazzari P.L., Alviano F., Ricci F., Capelli I., Cuna V., Todeschini P., Brunocilla E., Pagliaro P. (2014). Neutrophil gelatinase-associated lipocalin increases HLA-G^+^/FoxP3^+^ T-regulatory cell population in an *in vitro* model of PBMC. PLoS ONE.

[25] Rodvold J.J., Mahadevan N.R., Zanetti M. (2012). Lipocalin2 in cancer: When good immunity goes bad. Cancer Lett..

[26] Sultan S. (2012). Serum Lipocalin-2 (LCN-2) as a Major Acute Phase Protein under Different Pathological Conditions: *In vivo* and *in vitro* Studies. Ph.D. Thesis.

[27] Malik I.A., Moriconi F., Sheikh N., Naz N., Khan S., Dudas J., Mansuroglu T., Hess C.F., Rave-Fränk M., Christiansen H. (2010). Single-dose γ-irradiation induces up-regulation of chemokine gene expression and recruitment of granulocytes into the portal area but not into other regions of rat hepatic tissue. Am. J. Pathol..

[28] Ramadori G., Moebius U., Dienes H.P., Meuer S., Meyer zum Buschenfelde K.H. (1990). Lymphocytes from hepatic inflammatory infiltrate kill rat hepatocytes in primary culture. Comparison with peripheral blood lymphocytes. Virchows Arch. B Cell. Pathol. Incl. Mol. Pathol..

[29] Ramadori P., Ahmad G., Ramadori G. (2010). Cellular and molecular mechanisms regulating the hepatic erythropoietin expression during acute-phase response: A role for IL-6. Lab. Investig..

[30] Laemmli U.K. (1970). Cleavage of structural proteins during the assembly of the head of bacteriophage T4. Nature.

[31] Song B., Zhang H., Jiang L., Chi Y., Tian J., Du W., Yu B., Han Z. (2015). Down-regulation of lipocalin2 suppresses the growth of human lung adenocarcinoma through oxidative stress involving Nrf2/HO-1 signaling. Acta Biochim. Biophys. Sin. (Shanghai).

